# Electrical response of liquid crystal cells doped with multi-walled carbon nanotubes

**DOI:** 10.3762/bjnano.6.39

**Published:** 2015-02-06

**Authors:** Amanda García-García, Ricardo Vergaz, José Francisco Algorri, Xabier Quintana, José Manuel Otón

**Affiliations:** 1 CEMDATIC, E.T.S.I. Telecomunicación, Universidad Politécnica de Madrid, Avda. Complutense 30, Madrid, E28040, Spain; 2 GDAF-UC3M, Departamento de Tecnología Electrónica, Universidad Carlos III de Madrid, Butarque 15, Leganés, E28911, Spain

**Keywords:** carbon nanotubes, Cole–Cole diagrams, impedance, liquid crystal, PEDOT:PSS

## Abstract

The inclusion of nanoparticles modifies a number of fundamental properties of many materials. Doping of nanoparticles in self-organized materials such as liquid crystals may be of interest for the reciprocal interaction between the matrix and the nanoparticles. Elongated nanoparticles and nanotubes can be aligned and reoriented by the liquid crystal, inducing noticeable changes in their optical and electrical properties. In this work, cells of liquid crystal doped with high aspect ratio multi-walled carbon nanotubes have been prepared, and their characteristic impedance has been studied at different frequencies and excitation voltages. The results demonstrate alterations in the anisotropic conductivity of the samples with the applied electric field, which can be followed by monitoring the impedance evolution with the excitation voltage. Results are consistent with a possible electric contact between the coated substrates of the LC cell caused by the reorientation of the nanotubes. The reversibility of the doped system upon removal of the electric field is quite low.

## Introduction

Carbon-based nanostructured materials and their relationship with liquid crystals (LC) is a hot topic in current research. It is worth mentioning the recently described connection between graphene oxide and liquid crystals [1–2], as well as the highly active topic of LC structures doped with carbon nanotubes (CNTs) and the possibility of reorienting them with external fields [3–6]. The interest to control this reorientation arises from the possibility of preparing simple devices whose electrical conductivity can be externally controlled and modulated [5–9].

Due to their outstanding physical properties, CNTs have attracted a great deal of interest during the past 25 years [3–6]. They are formed from one or several rolled-up graphene sheets. CNTs show peculiar electrical properties: In single-walled carbon nanotubes (SWCNT), the conductivity is either metallic or semiconductive, while in multi-walled CNTs (MWCNT) it is always metallic [10]. The almost one-dimensional structure leads to a long-range ballistic electron transport in metallic CNTs [10–11].

LCs are self-organized anisotropic fluids, whose long-range orientation (called director) can be induced by surface conditioning of the cell walls [12] and modified by application of external electric fields above a certain voltage called Freedericksz threshold. Depending on the applied voltage, the LC dielectric permittivity along the electric field varies since the LC director adopts a specific orientation in order to minimize the energy derived from the electric field and the anchoring elastic forces [13].

These LC properties may be used to induce alignment and reorientation on dispersed CNTs. Using this effect, several photonic and electronic devices have been proposed [5] including electrically controlled switches. Remarkable conductivity differences between CNT-doped and undoped LC cells have been reported, and studies about variations in the dielectric permittivity [14–15], threshold voltage [16] and response time [17–18] have been published. Yet a more detailed description of the electrical behavior of CNT-doped LC cells under different conditions seems to be missing. This could be achieved by studying the impedance evolution of the doped cells with voltage and frequency. Indeed, the electrical response may be fairly complex since dielectric and anisotropic conductive elements are present in the system. The usual way to deal with these mixed elements is to model them with an equivalent electrical circuit (EEC). Once the cell behavior has been translated to elementary electrical elements (resistors, capacitors, inductors, etc.), the contribution of each component – LC, CNTs, external circuitry – to the electrical parameters can be established.

This work intends to describe CNT-doped LC cells by means of an EEC. Instead of designing a complicated multicomponent EEC, a simple EEC already employed to describe undoped cells will be tested. The validity of the EEC will be checked using CNTs as conductive elements, making it possible to create electrical interconnections between the external electrodes of the LC cells, the connections being controlled by the applied driving voltage.

## Electrical description of the system

**Figure 1:**
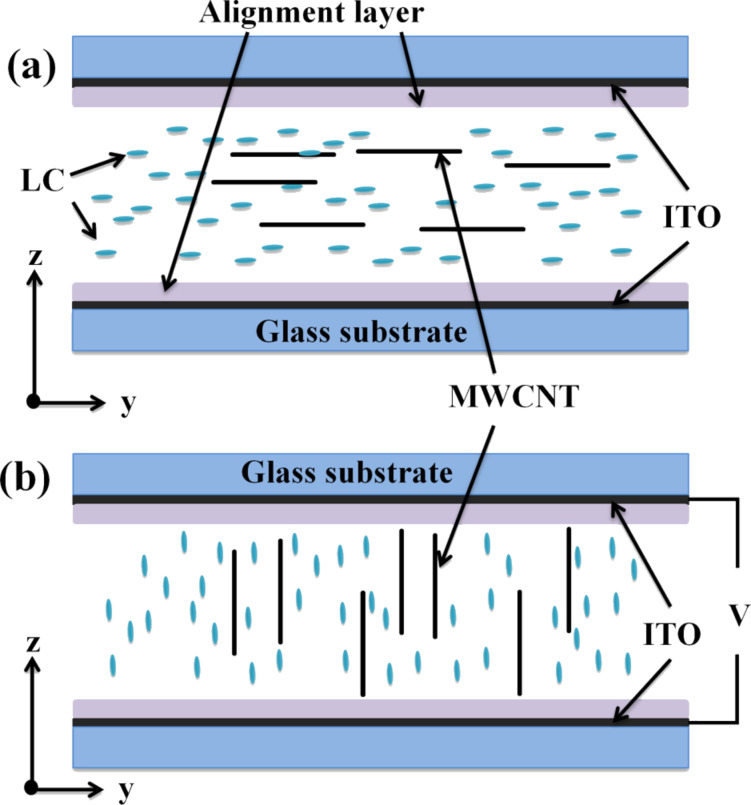
Schematic structure of a positive nematic MWCNTs-doped LC cell (a) without excitation voltage – planar orientation – and (b) at saturation voltage – vertical orientation.

Figure 1 resumes the hypothesis that this work expects to confirm. The sketch is not to scale: CNTs are several orders of magnitude longer than LC molecules, while their width is only 2–3 times as much. In a typical configuration, an MWCNT-doped LC with positive electric anisotropy is oriented homogeneously (i.e., parallel to the outer plates). Applying a saturation voltage between the electrodes coated onto the inner surfaces of the plates, the LC and presumably the MWCNTs reorient to adopt a homeotropic (i.e., perpendicular) configuration. If the thickness of the cell is similar to the MWCNT length, the conductive properties of the MWCNT should generate a noticeable variation of the electrical conductivity.

An apparently obvious condition for this effect to be detected is that electrical continuity must be kept across the LC cell. The alignment layers deposited on the plates to induce the LC orientation are usually made of non-conductive organic polymers. This issue has not always been taken into account in previous works [5,7], and may lead to ambiguous results. In this work, conductive alignment layers have been employed.

Several equivalent electrical circuits have been reported [19–20] describing the behavior of LC cells under different circumstances. In our case it is intended to employ a simple EEC where every contribution of LC, CNTs, and electric circuitry may be associated, if possible, to a single component. It is possible to achieve a good description of the cell with such a simplified model providing the frequency range is restricted to mid-range values, e.g., from 100 Hz to 10 kHz.

The actual measurements carried out in this work range from 100 Hz to 10 MHz. However, it has been observed that adjusting the highest frequency zone requires a far more complex EEC containing up to eight components. Although this circuit has been obtained and correctly adjusted to experimental data, the physical meaning of every component is occasionally questionable. Restricting the frequency range, however, makes matching between cell elements and circuit components straightforward. This need not to be a disadvantage, since LC cells are usually driven in these frequency ranges [21]: lower frequencies lead to flickering from LC reorientation following the electric field, while higher frequencies may affect the dielectric anisotropy [13].

**Figure 2:**
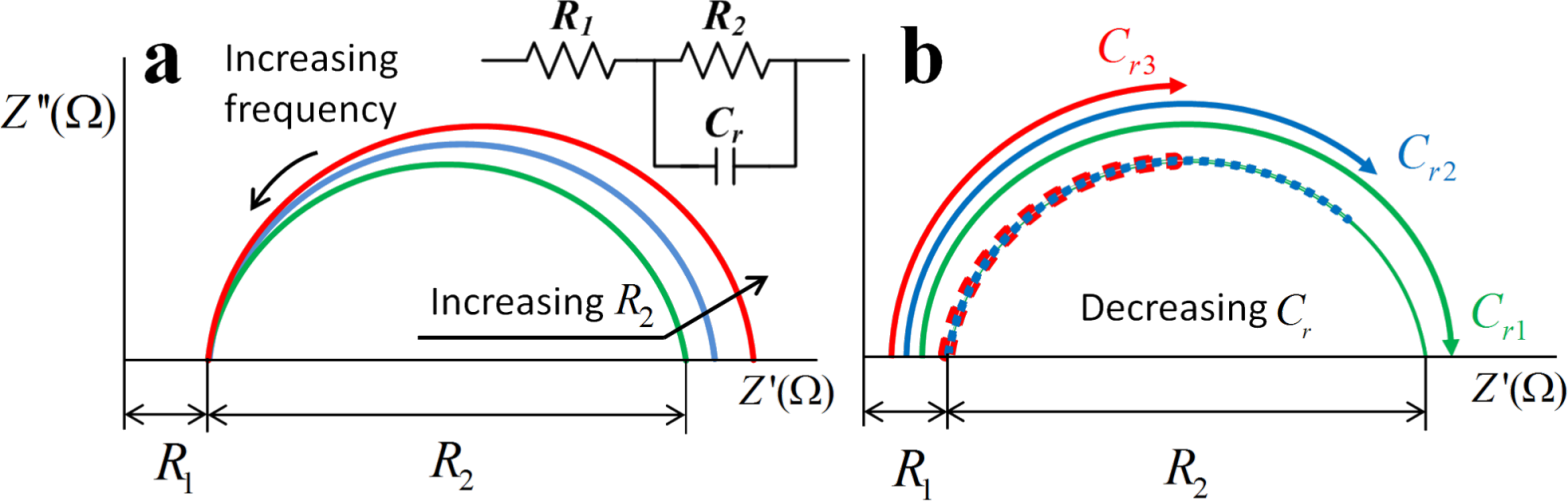
Sketch of frequency (a), resistance (a) and capacity (b, where *C_r_*_1_ *< C_r_*_2_ *< C_r_*_3_) variations in the Cole–Cole plot of the proposed EEC.

Within this restricted frequency range, LC cells may be considered to have a simplified EEC consisting of a resistor in series with the parallel set of a capacitor and a resistor (Figure 2) [22]. Every circuit element can be matched to physical parameters as follows:

- *R*_1_ stands for the resistivity of the outer elements and electrodes connections.
- *R*_2_ results from the conductivity of the doped or undoped LC material and is caused by the mobility of free charges, dipolar displacement and MWCNT conductivity.
- *C_r_* represents the device capacitance associated to the dielectric response.

The resulting impedance *Z*(ω) of the EEC can be expressed as follows:

[1]
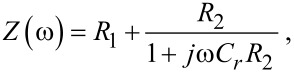


where ω = 2π*f*. By using low resistance indium tin oxide (ITO) electrodes (100 Ω/□, ohms per square, in our case) in the fabrication process, *R*_1_ is in the order of a few hundred ohms. Then, the circuit should be dominated by the parallel of *R*_2_ and *C_r_*. Equation 1 can be rewritten to identify the real (*Z*′) and the imaginary (*Z*″) part of *Z*(ω):

[2]
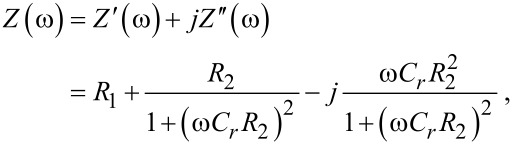


[3]
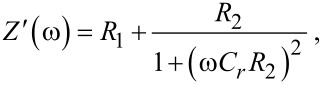


[4]
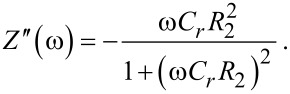


Depending on the frequency range and the magnitudes of *R*_2_ and *C_r_* the impedance of the system may exhibit a predominantly resistive or capacitive behavior. Note that the imaginary impedance is expected to be negative all over the range.

For undoped cells, the dielectric nature of the LC layer makes the capacitor the usually predominating element in the above mentioned operating frequency range, from 100 Hz to 10 kHz. As *C*_r_ is proportional to the dielectric permittivity, its value is expected to vary with the applied voltage upon reorientation of the material, due to the dielectric anisotropy of LC. For MWCNT-doped LC cells, one could also expect a change in the system conductivity because MWCNTs are conductive elements. If the conductivity increases, *R*_2_ could decrease significantly, causing a resistor-dominated impedance at least at low frequencies, because the impedance of *R*_2_ is lower than that of *C_r_*. This decrease in *R*_2_ should be more significant depending on the degree of reorientation of the MWCNTs.

The impedance behavior of MWCNT-doped and undoped LC cells has been studied by impedance spectroscopy. The results are shown in Cole–Cole plots of the imaginary (*Z*″) versus the real part (*Z*′) of the impedance. In these plots, also known as Nyquist plots, a resistor is simply a dot while an ideal capacitor is a vertical straight line. Computing *Z*′ and *Z*″ values at different frequencies, the outcome of this EEC is a semicircle, with diameter *R*_2_, which intercepts the x-axis on the right of *R*_1_ (Figure 2, note that –*Z*″ is customarily plotted, see Equation 4). The point ω = 0 is on the right side of the plot (*Z*′(ω = 0) = *R*_1_* + R*_2_ since the capacitor is an open circuit, see Equation 3). The frequency increases towards lower *Z’* values. Increasing the capacity of *C_r_*, while keeping a constant frequency range, impedes the completion of the semicircle (Figure 2, right).

## Results and Discussion

### Sample thickness and phase shift

**Figure 3:**
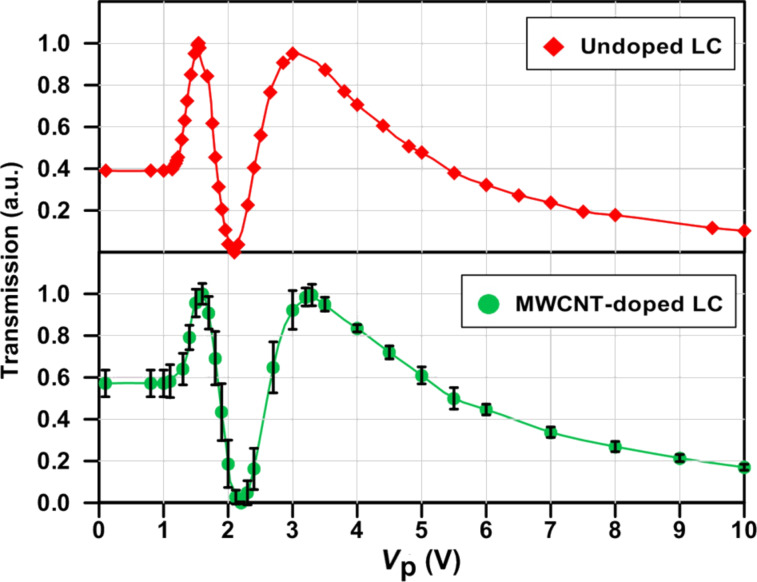
Light transmission response of undoped (top) and MWCNT-doped (bottom) cells at 543 nm. The bottom plot is an average of three samples.

One cell of each batch was tested for LC reorientation and thickness. Cells were placed between crossed polarizers with the optical indicatrix oriented at 45°, and driven with a 1 kHz square signal of varying amplitude. The probing wavelength was 543 nm, close enough to the 588 nm wavelength employed in the LC data sheet for birefringence measurement. Figure 3 is an example of the results. From the saturating side *V* ≥ 8 V_p_ (where p indicates the “peak voltage”, see Experimental section) down to the maximum at about 1.8 V_p_, the phase delay is δ = 3π. From 1.8 to 1.3 V_p_ (the threshold voltage), the delay increases further, about 2π/3 according to the sine value. Using the value Δ*n* = 0.120 of the data sheet yields

[5]
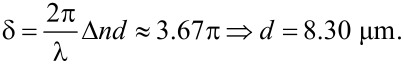


The cell thickness matches very well with the expected manufacturing value (8.25 μm). Similar results have been obtained in all cases. Therefore, the thickness is assumed to be constant, and threshold and saturation voltages are set to 1.3 V_p_ and 8 V_p_, respectively. The cells employed in these tests are not further used in the impedance experiments.

With this information, the selected bias voltage amplitudes – actually, the peak-voltage of the low-frequency square signal – were 0 V_p_ (planar alignment), 3 V_p_ (an intermediate state between threshold and saturated state) and 8 V_p_ (perpendicular orientation). The reversible character of the switching was monitored by following a predetermined bias voltage sequence: once the cells had been driven at 3 V_p_ or 8 V_p_, a new scan at 0 V_p_ was acquired. An identical result at all 0 V_p_ measurements would confirm a reversible operation mode.

### Impedance of unbiased/biased and undoped/doped samples

**Figure 4:**
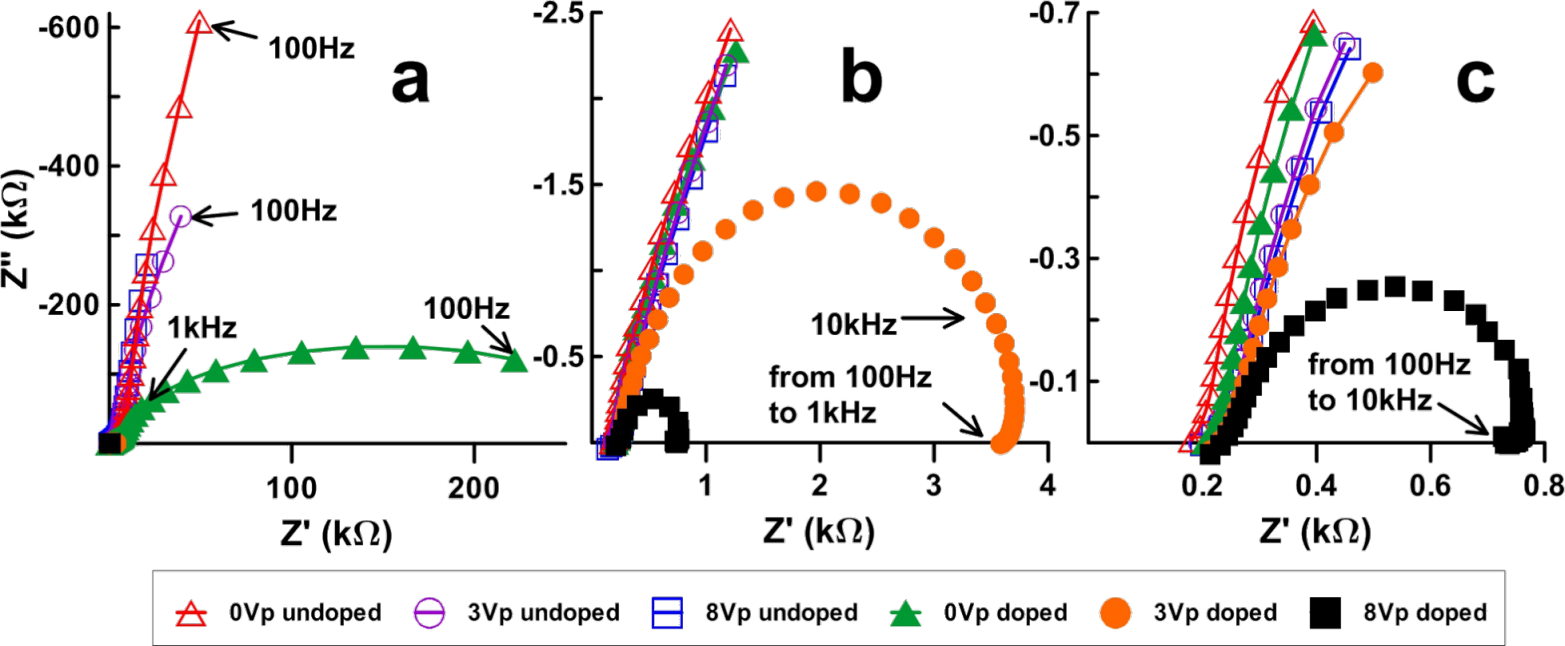
Cole-Cole plots of the undoped and MWCNTs-doped LC cells at different scales. (a), (b) and (c), impedance evolution with the reorientation of LC molecules (0, 3 and 8 V bias voltages).

Figure 4a–c show the impedance results of undoped and MWCNT-doped LC cells with different bias voltages (i.e., 3 + 3 curves) arranged in Cole–Cole plots. The three graphs intend to show the same results at different XY-axes scales: Figure 4b is an enlargement of Figure 4a, and Figure 4c shows even more details. This sequential zoom-in is essential for the inspection of quite dissimilar details being noticeable at different scales.

In undoped LC cells, measurements confirm a capacitor effect dominating at mid-range frequencies; the capacitance prevails for all polarization voltages and frequencies (Figure 4a). Nevertheless, an ideal capacitor should appear as a vertical straight line in the Cole–Cole plot. The tilts of the lines indicate a small contribution of the resistive components, i.e., capacitor losses in the dielectric. Lines become more tilted (i.e., less vertical) as the bias voltage increases. The reorientation of LC increases the dielectric permittivity as "seen" by the applied electric field. Consequently, the cell capacity increases and the capacitance decreases (as seen in Figure 2). Slight deviations from a straight line can be seen at high frequencies in the enlarged Figure 4c.

The electrical behavior of MWCNT-doped LC cells differs significantly from this result. The measurement of the doped cells 0 V_p_, as seen in Figure 4a, shows a remarkable decrease of the impedance along with a much larger contribution of the resistive component *R*_2_. In other words, the mere presence of MWCNTs modifies significantly the electric behavior of the unswitched LC sample. The undoped LC cell is basically a capacitor (i.e., *R*_2_ → ∞) while the doped cell behaves as a capacitor in parallel with a resistor. Although the ohmic resistance is high (several hundred kiloohms), the most probable explanation is that a small fraction of MWCNTs is not oriented as the liquid crystal, but in tilted positions through which some electric paths between the plates can be established. The presence of ionic impurities in the MWCNTs might somehow contribute to the conductivity as well; however, the extremely low concentration of CNTs makes it rather improbable that these ions play any significant role in the sample conductivity. The curve is nearly a semicircle, the peak *Z*″ value coinciding with *R*_2_/2, i.e., the vertical and horizontal radii. Note that the whole semicircle is developed in the case at medium frequencies (from 100 Hz to 1 kHz), in which the simplified EEC model applies.

The conductivity increases dramatically if voltages above threshold (Figure 4b) or above saturation (Figure 4c) are applied. Note that these curves are just points in Figure 4a. The DC (ohmic) resistance (i.e., the *R*_2_ value) is about 2 kΩ when the applied voltage is above the threshold (Figure 4b) and about 500 Ω when the applied voltage is above saturation (Figure 4c). This remarkable decrease in the resistance can only be explained by a reorientation of the MWCNTs, creating multiple electrical paths between the electrodes.

It is worth mentioning that the experimental semicircles corresponding to 3 V_p_ and 8 V_p_ curves are biased. The right horizontal intercepts, corresponding to ω = 0 Hz, show a curvature towards lower values, while the imaginary *Z*″ peak is shifted to higher *Z*' (i.e., lower frequency values). Neither of these effects can be explained within the simple model employed in this work, since more electronic components are required for the EEC to fit these features which, anyhow, do not invalidate the above conclusions since they are fairly small.

### Reversibility of switched MWCNT cells

The switching reversibility has been tested by running several batches of MWCNT-doped samples with the following sequence: fresh unbiased samples (i.e., with 0 V polarization) were measured and then driven above threshold (3 V_p_). The unbiased samples were measured again and then driven above saturation (8 V_p_). A new unbiased scan was acquired afterwards. The sequence intends to check if MWCNTs reversibly return to their original orientation after being driven.

**Figure 5:**
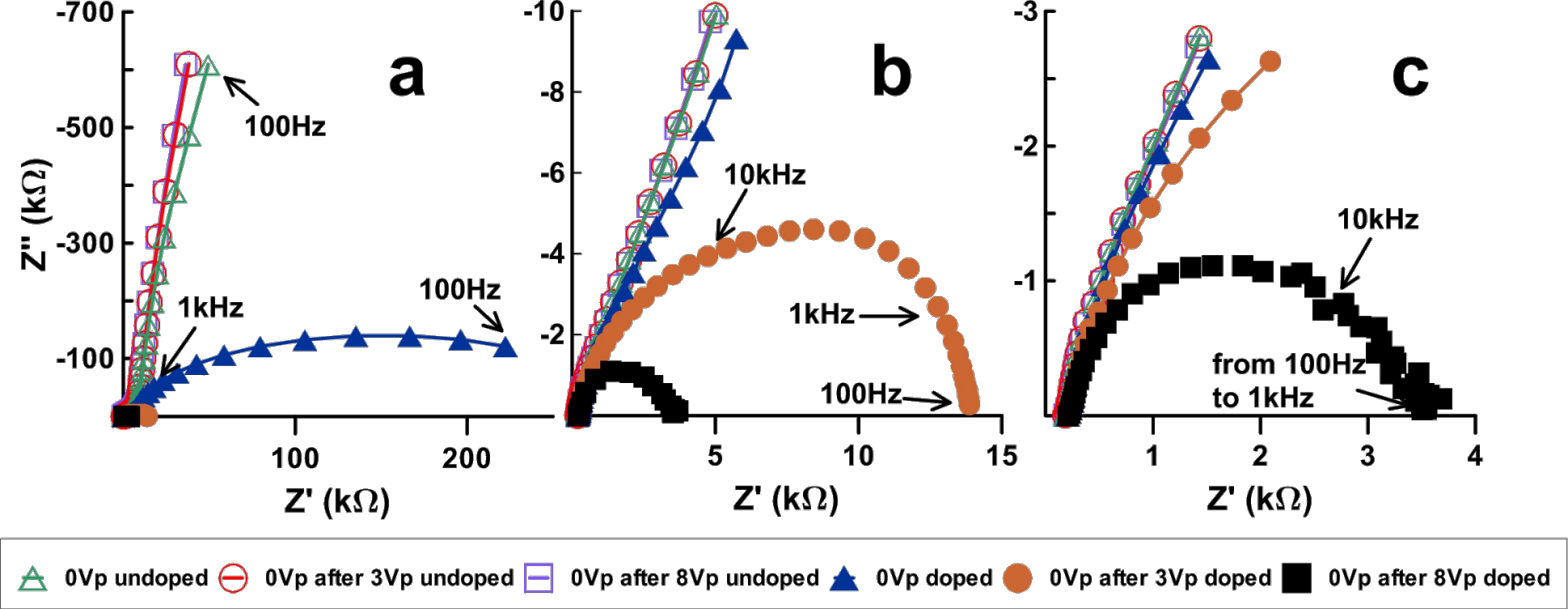
Impedance of unbiased doped (filled symbols) and undoped (unfilled symbols) samples at different instances of the driving sequence: initial 0 V, 0 V after *V*_p_ = 3 V and 0 V after *V*_p_ = 8 V. (a), (b) and (c) are different scales of the same graph.

The sequence results are summarized in Table 1. Figure 5a–c shows one of the batches, the enlargement steps being the same as in Figure 4. Table 1 is organized as follows: *f* is the frequency and *Z*' is the real impedance for which the maximum of |*Z*″| is obtained. *R*_1_ and *R*_2_ have been obtained from the EEC model by fitting the experimental results and the parameters through a Levenberg–Marquardt algorithm [23] employing the software package ZView (Scribner) version 3.3f. An extra column has been added for comparison purposes: Through geometrical considerations (see Figure 2), Equation 6 can be derived from the EEC model:

[6]
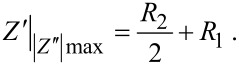


The results are shown side by side as an indication on whether the simple EEC model employed in this work holds.

**Table 1:** Peak values of *Z*" and comparison of *Z*' values with computed *R*_1_*, R*_2_ (average of three batches).

driving sequence	*f* (kHz)	|*Z”*| (kΩ)	*Z*′ (kΩ)^a^	*R*_2_/(2 + *R*_1_) (kΩ)	*R*_2_ (kΩ)	*R*_1_ (Ω)

0 V	0.29 ± 0.18	103.0 ± 39.8	113.0 ± 45.7	110.07	204.36 ± 75	7887 ± 572
3 V_p_	26.8 ± 4.3	1.54 ± 0.08	2.20 ± 0.20	2.13	3.68 ± 0.23	289 ± 19.0
0 V after 3 V_p_	4.55 ± 0.83	5.06 ± 0.42	8.88 ± 0.56	7.50	14.46 ± 0.89	272 ± 15.3
8 V_p_	189.8 ± 17.2	0.28 ± 0.02	0.58 ± 0.05	0.56	0.54 ± 0.08	292 ± 6.8
0 V after 8 V_p_	84.1 ± 14.1	1.17 ± 0.05	1.61 ± 0.17	1.96	3.36 ± 0.20	274 ± 1.5

^a^Value at |*Z*″| maximum.

Looking at the results for *R*_1_, all cases show approximately the same value except the original unbiased cells. According to the EEC model, *R*_1_ is external to the cell, and should be assigned to the contacts and the ITO layer that makes the electric contacts within the cell. Therefore, it should be constant along the sequence in all cells. The adjusted value, 270–290 Ω, fits well with the geometry of the cell and the specific resistance of ITO. The result obtained for the fresh unbiased cells, on the contrary, is not acceptable. This deviation is attributed to the large span scanned by these particular cells. These samples have a large capacitor component, their ohmic resistance being in the hundreds of kiloohms. In spite of the large error in *R*_1_, the left and right terms of Equation 6 fit reasonably well. Anyhow, it has been found in our simulations, according to [22], that the actual origin of the deviation arises from the presence of another semicircle (another electronic component) in the high frequency range above 10 kHz, i.e., in which the simple EEC model used here does not hold.

**Figure 6:**
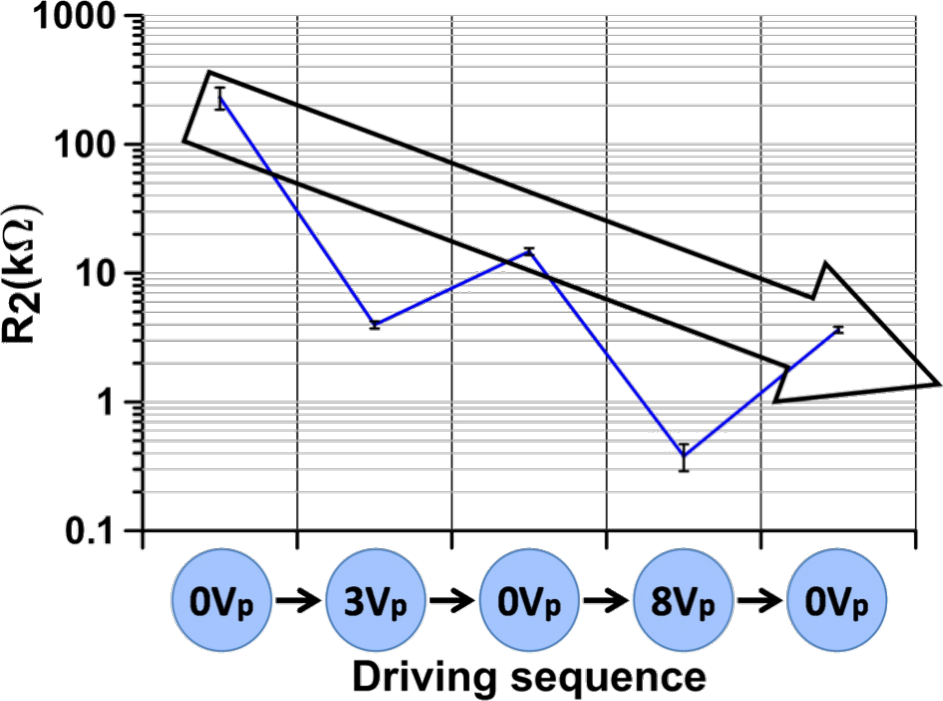
*R*_2_ average evolution with the driving sequence. The three unbiased averaged measurements are shown inside the arrow.

Besides the deviations of the unbiased cells, the most important result of this series is the low reversibility of the doped system. Once the cells are driven above threshold, the impedance at 0 V_p_ reduces dramatically compared to fresh undriven cells (Figure 6). Compared to biased (3 V_p_) results, the impedance at 0 V_p_ after driving increases only slightly (about four times) and the frequency decreases accordingly (to about one sixth). In other words, the impedance at 0 V_p_ remains close to the biased measurement rather than turning back to its original value. No significant differences have been found between cells measured 15 min after driving and longer times up to one week.

The same results in a more pronounced manner are obtained when the cells are driven above saturation. Apparently, MWCNTs are reoriented by electric fields as if they were following the LC reorientation. However, they are almost unable to follow the LC upon relaxation. It can be derived that applying an electric field induces permanent modifications on the MWCNT dispersion.

There are several causes that might explain this behavior and they are currently under study. The nanotubes contain charges and ionic impurities that could contribute to the MWCNT reorientation with applied field, but do not contribute to the eventual relaxation. A second cause that could contribute to the effect is the π–π stacking between CNTs and LC molecules [24], generating a hybrid structure that follows the electric field, but does not relax as the restoring elastic forces cannot compete with nanometer-sized structures. This idea is supported by the fact that the high resistivity of the original state is not restored even by heating the sample above the LC isotropic transition point and subsequent slow cool down. The LC apparently recovers the original orientation, although contrast is impaired, but the electric response remains approximately the same. Moreover, electrical anisotropic behavior has been found in CNT-doped LC cells above the isotropic transition [14]. The authors explain these results by assuming a strong anchoring between the LC molecules and the CNTs. This anchoring keeps the former arranged in pseudo-nematic oriented domains around the latter above the isotropic point. Confirmation of this phenomenon and other features of the CNT–LC system are currently been checked in our laboratory by Mach–Zehnder interferometry, Raman spectroscopy, and additional impedance measurements. This should be the focus of future research.

## Conclusion

A procedure to prepare MWCNT-doped LC cells with electrical continuity between the outer electrodes has been developed and their impedance has been studied and compared to undoped LC cells. For MWCNT-doped LC cells, the measurements reveal dominant resistor behavior at mid-range frequencies. The impedance magnitude decreases with the voltage applied to the doped LC cell, and the frequency range at which the resistor behavior is dominating increases. The effect is not reversible as the resistor behavior persists when the exciting voltage is brought back to values below the threshold voltage.

## Experimental

### Materials: LCs and MWCNTs

The LC MLC-6290-000 (Merck) has been chosen for this study. It is a well-known positive dielectric nematic mixture that has been used in low-end LC displays. Its main features are a kinematic viscosity of 20 mm^2^·s^−1^ at 20 °C and an optical birefringence (Δ*n*) of 0.120 at 588 nm. The material shows a wide nematic phase range above and below room temperature.

The CNTs employed in the experiments have been MWCNTs from Sigma-Aldrich. These MWCNTs, according to the data sheet, have a tube length of about 5 µm and an outer tube diameter of 6–9 nm. Therefore, their aspect ratio is very high (approximately 1000:1).

### Preparation of CNT-doped LC mixtures

Preliminary experiments established that the most interesting range of MWCNT concentrations in LC–MWCNT mixtures was about 0.01 wt %. All the experiments have been realized at this concentration. Given that a reduced MWCNT amount had to be used in each batch, a number of successive MWCNT dilutions were carried out first in toluene (down to 0.1 wt %) and in the LC itself afterwards. The samples were heated over the LC isotropic transition and sonicated for 30 min to achieve homogeneous mixtures and to evaporate the remaining toluene.

#### Sample configuration

0.7 mm thick ITO-coated glass plates (100 Ω/□) from Glasstone were used. The cell thickness was 8.25 µm (more than the length of the MWCNTs). An active area of 1 cm^2^ was defined through photolithography by removing the ITO electrode of the outer surface. The LC alignment configuration was planar, i.e., with the LC director oriented parallel to the plane of the electrode plates at the off-state. The planar LC alignment was induced by PEDOT:PSS [poly(3,4-ethylenedioxythiophene) poly(styrenesulfonate)]. A 1.3 wt % solution of PEDOT:PSS in water (Sigma-Aldrich) was spin-coated onto the plates and eventually buffed with a velvet cloth to induce a specific planar orientation.

As mentioned above, other polymers such as polyimide are preferred as alignment layers for these LC devices. However, preliminary work with polyimide alignment layers showed that these effectively act as isolating layers, hindering eventual MWCNT-derived conductivity. Despite being a much less efficient aligning surface, PEDOT:PSS has a remarkably higher conductivity than polyimide, what is essential to keep electric continuity across the layer.

#### Characterization method: driving waveform

**Figure 7:**
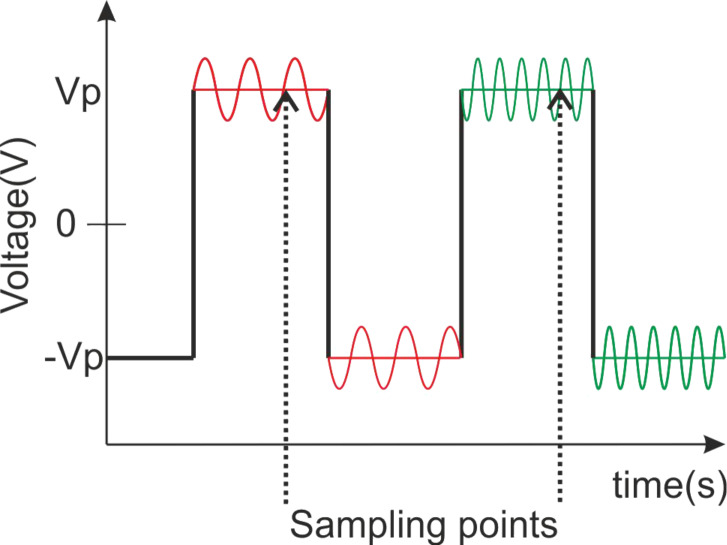
Driving waveform for impedance measurements. *V*_p_ is peak voltage.

Impedance spectroscopy customarily employs sufficiently small voltage signals so that the system response is linear. If the study includes the effect of external electric fields, as in this case, the AC signal probe should be set on a bias (offset) DC voltage. However DC voltage leads to electrolytic degeneration of the LC cell by ion generation and migration, and eventual adsorption of the charges onto the alignment layers. To avoid this issue, DC bias has been substituted by a low frequency (0.5 Hz) AC square wave to which the low amplitude AC probe voltage signal is added up (Figure 7). Being a square signal, reorientation of the LC is minimally affected by polarity changes. However, sampling is performed near the end of every cycle to allow the LC to stabilize further. Data from positive and negative half-cycles were separately collected to check for deviations from each other. No significant deviations were found.

Three different square voltage amplitudes, *V*_peak_ = 0 V_p_, 3 V_p_, 8 V_p_, have been used. These correspond (see Figure 3) to voltage levels below the Freedericksz threshold, to an intermediate value, and to a value close to saturation, respectively. A sinusoidal signal of 100 mV_rms_ was added up in all cases, its frequency was varied from 100 Hz up to 10 MHz. The waveform was programmed in a 1260 Solartron impedance analyzer controlled by a PC that collects data as well.
